# A review of *Aulacospira* Möllendorff, 1890 and *Pseudostreptaxis* Möllendorff, 1890 in the Philippines (Gastropoda, Pupilloidea, Hypselostomatidae)

**DOI:** 10.3897/zookeys.842.33052

**Published:** 2019-05-07

**Authors:** Barna Páll-Gergely, Menno Schilthuizen, Aydin Örstan, Kurt Auffenberg

**Affiliations:** 1 Plant Protection Institute, Centre for Agricultural Research, Hungarian Academy of Sciences, Budapest 1022, Herman Ottó út 15, Hungary Centre for Agricultural Research, Hungarian Academy of Sciences Budapest Hungary; 2 Naturalis Biodiversity Center, Darwinweg 2, 2333 CR Leiden, The Netherlands Naturalis Biodiversity Center Leiden Netherlands; 3 Institute for Biology Leiden, Leiden University, Sylviusweg 72, 2333 BE Leiden, The Netherlands Leiden University Leiden Netherlands; 4 Institute for Tropical Biology and Conservation, Universiti Malaysia Sabah, Jalan UMS, 88400 Kota Kinabalu, Sabah, Malaysia Universiti Malaysia Sabah Kota Kinabalu Malaysia; 5 Research Associate, Section of Mollusks, Carnegie Museum of Natural History, Pittsburgh, Pennsylvania, USA Carnegie Museum of Natural History Pittsburgh United States of America; 6 Florida Museum of Natural History, University of Florida, Gainesville, Florida, USA University of Florida Gainesville United States of America

**Keywords:** taxonomy, shell, systematics

## Abstract

The genera *Aulacospira* and *Pseudostreptaxis* of the Philippines are revised based on the collections of the Senckenberg Forschungsinstitut und Naturmuseum (Frankfurt am Main, Germany), the Florida Museum of Natural History (USA), and recently collected material. Three new species are described: *Aulacospiralens* Páll-Gergely & Auffenberg, **sp. n.**, *Aulacospirakrobyloides* Páll-Gergely & Schilthuizen, **sp. n.**, *Pseudostreptaxisharli* Páll-Gergely & Schilthuizen, **sp. n.**

## Introduction

The genus *Aulacospira* Möllendorff, 1890 is represented by six species in the Philippines: *A.hololoma* (Möllendorff, 1887), *A.mucronata* (Möllendorff, 1887), *A.scalatella* (Möllendorff, 1888), *A.porrecta* Quadras & Möllendorff, 1894, *A.rhombostoma*Quadras & Möllendorff, 1896, and *A.triptycha* Quadras & Möllendorff, 1895. Möllendorff (1890) described the monotypic genus *Pseudostreptaxis* for Helix (Aulacospira) azpeitiae Hidalgo, 1890 as a section of *Aulacospira*. Historically, *Aulacospira* is reported only from the Philippines. Recently, new species of the genus were described from Thailand (Panha and Burch 2001, 2005, Dumrongrojwattana and Panha 2005, 2006, Dumrongrojwattana 2008) and Vietnam (Vermeulen et al. 2007). This unusual distributional pattern is most probably due to convergence of shell shape. This phenomenon was revealed by the molecular phylogeny of Thai Hypselostomatidae (Tongkerd et al. 2004). Species inhabiting the Philippines are probably not closely related to those of the Asian continent.

Here we provide an overview of the Philippine *Aulacospira* and *Pseudostreptaxis* and describe two new species of the former and one new species of the latter. The two groups are treated as full genera of their own right following Schileyko (1998).

## Materials and methods

Shell whorls (± 0.25) were counted according to Kerney and Cameron (1979: 13). Shells were measured using a vernier caliper. Photographs of specimens deposited in the SMF were taken using a Nikon camera and a macro lens, whereas those of the new species were photographed with a Keyence LHX5000 digital microscope. In both cases 5–20 photos were taken of each shell, and merged to create a single image using Photoshop.

**NHMW** Naturhistorisches Museum Wien (Vienna, Austria)

**PGB** Collection Barna Páll-Gergely (Mosonmagyaróvár, Hungary)

**REI** Collection Reischütz (Horn, Austria)

**RMNH** Naturalis Biodiversity Center (Leiden, The Netherlands)

**SMF** Senckenberg Forschungsinstitut und Naturmuseum (Frankfurt am Main, Germany)

**UF** Florida Museum of Natural History (University of Florida, Gainesville, USA)

**coll.** collection of

**leg.** collected by

**ex.** from the collection of

**D** shell diameter

**H** shell height

## Taxonomy

### Family Hypselostomatidae Zilch, 1959

**Remarks.**Zilch (1959) erected Aulacospirinae (type genus: *Aulacospira* Möllendorff, 1890) and Hypselostomatinae (type genus: *Hypselostoma* Benson, 1856) as subfamilies of Chondrinidae. Gittenberger (1973) synonymized those two groups with Gastrocoptinae Pilsbry, 1918. Schileyko (1998) claimed that the characters of the two subfamilies overlap or are incomparable, and giving precedence to Hypselostomatinae over Aulacospirinae, he maintained them as a single group under Hypselostomatidae (Schileyko 1998: 136). Bouchet et al. (2017) accepted Gittenberger’s (1973) classification, and treated both Hypselostomatinae and Aulacospirinae as synonyms of Gastrocoptidae, noting that the relationships between those groups could not be solved by recent molecular phylogenetic research. Pilsbry (1916–1918) classified *Aulacospira* (and its subgenus, *Pseudostreptaxis*) into the subfamily Gastrocoptinae of the family Pupillidae. Vermeulen et al. (2007) treated *Aulacospira* as a vertiginid, whereas Thai authors (Panha and Burch 2001, 2005, Dumrongrojwattana 2008, Dumrongrojwattana and Panha 2005, 2006) treated it as a pupillid genus. Here we follow Schileyko (1998), and include *Aulacospira* Möllendorff, 1890 and *Pseudostreptaxis* Möllendorff, 1890 within the Hypselostomatidae.

#### Genus Aulacospira

Taxon classificationAnimaliaStylommatophoraHypselostomatidae

Möllendorff, 1890

Aulacospira Möllendorff, 1890: 224.
Micropetasus Möllendorff, 1890: 224 (Aulacospira sect.; type species: Helixscalatella Möllendorff, 1888.
Aulacospira Schileyko, 1998: 140.

##### Type species.

*Helixscalatella* Möllendorff, 1888, subsequent designation by Pilsbry (1895) [in Pilsbry 1894–1895]).

##### Diagnosis.

Shell triangular, low conical or lenticular, body whorl keeled, whorls sometimes scalariform, body whorl (sometimes only the last quarter whorl, sometimes all whorls) with subsutural furrow dorsally; protoconch smooth or spirally striated, teleoconch usually spirally striated and irregularly wrinkled; aperture adnate to penultimate whorl with weak parietal callus or free from penultimate whorl with continuous aperture; apertural barriers 0–5; umbilicus very narrow to moderately narrow.

#### Aulacospira hololoma

Taxon classificationAnimaliaStylommatophoraHypselostomatidae

(Möllendorff, 1887)

Figures 1D , 5E

Helix hololoma Möllendorff, 1887: 275, plate 8, figs 12–12b.
Aulacospira (Micropetasus) hololoma Möllendorff, 1890: 225.
Aulacospira hololoma Möllendorff, 1898: 150; Pilsbry 1917 (1916–1918): 222, plate 38, figs 6–7.
Aulacospira (Aulacospira) hololoma Zilch, 1984: 166, plate 2, fig. 23.

##### Type locality.

“in cacumine montis Licos insulae Cebu”.

##### Diagnosis.

Shell triangular, keeled, end of body whorl with slight subsutural furrow on the dorsal side, protoconch spirally striated, teleoconch roughly wrinkled and finely striated spirally; aperture with a single columellar tooth; aperture free from penultimate whorl.

**Figure 1.**
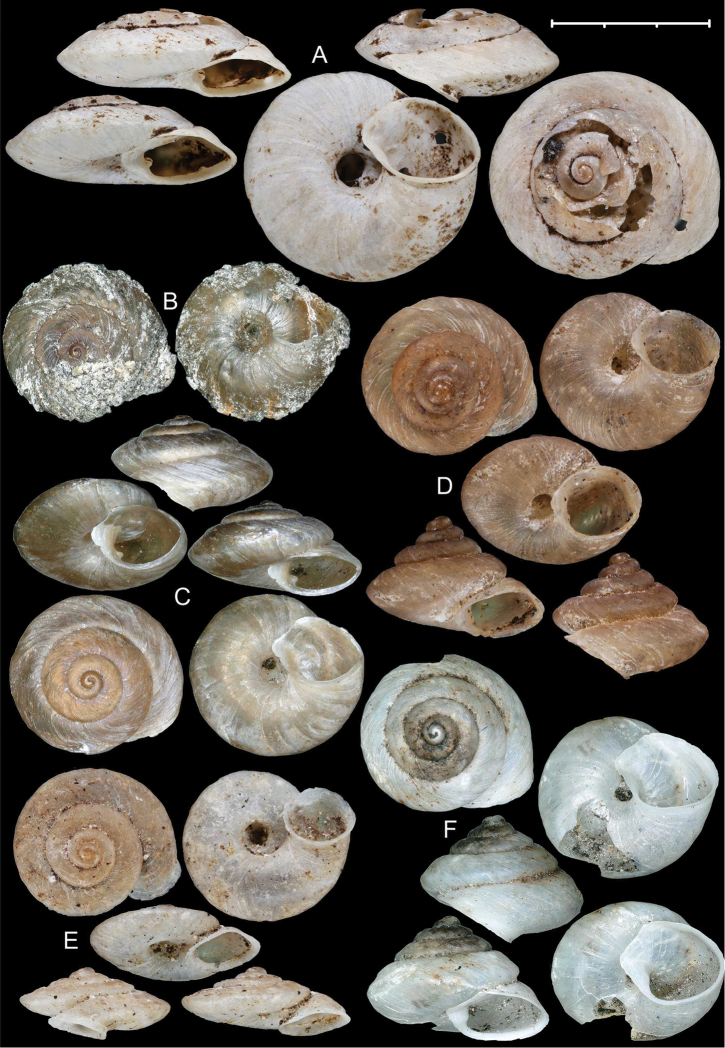
Shells of *Aulacospira* Möllendorff, 1890. **A***Aulacospiratriptycha* Quadras & Möllendorff, 1895 (SMF 4611, syntype) **B***Aulacospiralens* Páll-Gergely & Auffenberg sp. n. (paratype, UF 245505, uncleaned with original coating) **C***Aulacospiralens* sp. n. (UF 245485, holotype) **D***Aulacospirahololoma* (Möllendorff, 1887) (SMF 4608, lectotype) **E***Aulacospiramucronata* (Möllendorff, 1887) (SMF 4609, lectotype) **F***Aulacospirakrobyloides* Páll-Gergely & Schilthuizen sp. n. (RMNH.MOL.340281, holotype). Scale bar: 3 mm.

##### Measurements (in mm).

H = 2.3–2.5, D = 3.1–3.4 (n = 3).

##### Types examined.

Philippinen: Mte. Licos, Cebu, coll. Möllendorff, SMF 4608 (lectotype, selected by Zilch [1984], H: 2.4 mm, D: 3.3 mm); same data, SMF 10701/4 paralectotypes; same data, coll. O Boettger excoll. Möllendorff, SMF 63878/3 paralectotypes.

##### Additional material examined.

Cebu Id., leg. Univ. Alabama, TH Aldrich Coll., Ex: WF Webb (THA-3079), UF 112317 (4 shells).

##### Distribution.

**Figure 2.**
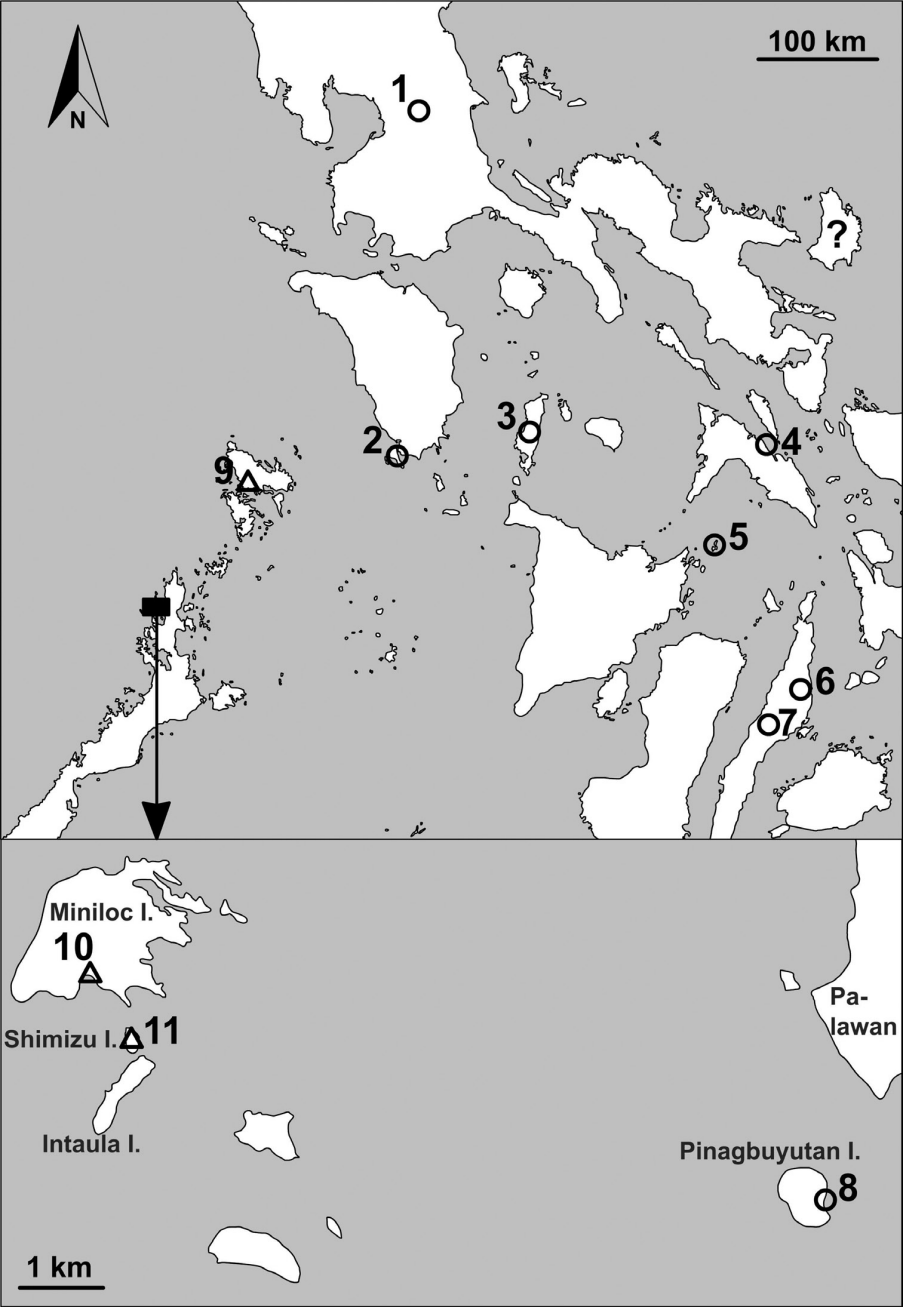
Distribution of *Aulacospira* (circles) and *Pseudostreptaxis* (triangles) species. The black square on the upper map is enlarged below. 1: *A.scalatella*; 2: *A.porrecta*; 3: *A.rhombostoma*; 4: *A.triptycha*; 5: *A.lens* sp. n., 6: *A.hololoma* and *A.mucronata*; 7: *A.mucronata*; 8: *A.krobyloides* sp. n.; 9: *P.azpeitiae*; 10–11: *P.harli* sp. n. Question mark shows Catanduanes Island, which is the type locality of *P.azpeitiae*.

This species is only known from the type locality on Cebu Island (Fig. 2).

#### Aulacospira krobyloides

Taxon classificationAnimaliaStylommatophoraHypselostomatidae

Páll-Gergely & Schilthuizen sp. n.

http://zoobank.org/B4054F63-6EFD-4C83-99D4-C40A704BCC08

Figures 1F , 5D

##### Type material.

Palawan Province, El Nido Municipality, Pinagbuyutan island, 11.121N, 119.394E, sieved from litter under limestone cliff, 17 Mar 2018, M Schilthuizen leg., TxEx-PA0001-17, RMNH.MOL.340281 (holotype, H: 2.4 mm, D: 3.5 mm).

##### Diagnosis.

Shell flattened and keeled, all whorls with slight subsutural furrow on the dorsal side, protoconch smooth with slight spiral striation, teleoconch roughly wrinkled and finely spirally striated; aperture with indication of a columellar tooth; aperture adnate to penultimate whorl.

##### Description.

Shell depressed-conical (ca. 1.5 times as wide as high) with a strong keel in the middle of body whorl, and a very slightly indicated subsutural furrow between the keel and the suture; colour off-white, but fresh specimens will probably have a darker colour; entire shell consists of 4.5 whorls; protoconch elevated, consisting of ca. 1.25 whorls, finely granulose, with some spiral striation; teleoconch finely granulose, with some fine, irregular wrinkles and fine, dense spiral striae; aperture strongly oblique to shell axis, aperture shape suboval; peristome slightly expanded from parieto-palatal junction to parieto-columellar junction, additionally slightly thickened between keel and columella, peristome slightly expanded in direction of umbilicus, partly covering it; parietal callus appears as additional, rather strong lime layer on parietal part; columellar tooth only indicated on columella, blunt; no parietal or palatal teeth present; umbilicus very narrow, shows all whorls, ca. its half is covered by expanded peristome edge.

##### Measurements (in mm).

H = 2.4, D = 3.5 (holotype).

##### Differential diagnosis.

*Aulacospirakrobyloides* sp. n. is most similar to *A.lens* sp. n., which is more depressed, has a wider umbilicus, lacks spiral striation on the teleoconch, and has a parietal tooth. *Aulacospirahololoma* has a higher conical shell, and its aperture is free from the preceding whorl. *Aulacospiratriptycha* is larger, flatter, has a wider umbilicus, and well-developed columellar tooth and a small palatal denticle.

##### Distribution.

This species is only known from the type locality on Pinagbuyutan Island (Fig. 2).

##### Etymology.

This new species is named for its resemblance to members of the genus *Krobylos* Panha & Burch, 1999.

#### Aulacospira lens

Taxon classificationAnimaliaStylommatophoraHypselostomatidae

Páll-Gergely & Auffenberg sp. n.

http://zoobank.org/E3D6E7DD-043B-4A37-8C27-D7368E764EF7

Figures 1B, C , 5F

##### Type material.

Panay Ids., Iloilo Prov., Carles Municipality, ca. 15.0 km E Carles, South Gigante Id., south shore, 75 m, 11°35'N, 123°20.5'E, 24 Apr 1992, K Auffenberg, et al. leg., KA-1039, UF 245485 (holotype, H: 2.0 mm, D: 3.5 mm); same data, UF 525638 (37 paratypes = juvenile/subadult shells); same locality, KA-1040, UF 245505 (7 paratypes = juvenile/subadult shells); same data, 9 paratypes in ethanol.

##### Diagnosis.

Shell strongly flattened and keeled, all whorls with slight subsutural furrow on the dorsal side, protoconch smooth with slight indication of spiral striation, teleoconch roughly wrinkled without spiral striation; aperture with a columellar and a parietal tooth; aperture adnate to penultimate whorl.

##### Description.

Shell strongly discoid (1.7–1.9 times as wide as high) with a strong peripheral keel, and a shallow subsutural furrow between the keel and the suture; colour light brownish-greyish with occasionally some purplish colouration; fresh shells with some sand or detritus attached to both dorsal and ventral surfaces, probably functioning as camouflage; shell consists of ca. 4 whorls; protoconch consisting of ca. 1.0 whorl, finely granulose, superficially smooth, with some very faint spiral striation; teleoconch finely granulose, with weak, irregular wrinkles, without spiral striae; aperture strongly oblique to shell axis, aperture shape suboval with palatal elongation due to keel; peristome sharp on the palatal portion above keel, slightly thickened and reflected between keel and columella, and slightly expanded toward umbilicus, partially covering it; parietal callus very weak, often transparent; columellar tooth low on the columella, well-developed, blunt; parietal lamella present only in holotype (all other shells are subadults), relatively long, low, slightly immersed from parietal callus; umbilicus narrow, showing all whorls, partially covered by expanded peristome.

##### Measurements (in mm).

H = 1.9–2.0, D = 3.5–3.6 (n = 2: the holotype and one large subadult shell).

##### Differential diagnosis.

*Aulacospiralens* sp. n. is similar to *A.hololoma* by the presence of a single columellar tooth, but differs from that species is being larger and much flatter. *Aulacospiramucronata* is similar to *A.lens* sp. n. in shape, but it differs from the new species by having a subsutural furrow on the dorsal side of the whorls, a comparatively smaller aperture, a wider umbilicus, and lacking any apertural barriers. *Aulacospiratriptycha* has a larger and flatter, spirally striate shell, a wider umbilicus, has no parietal tooth, but possesses a small palatal denticle.

##### Distribution.

This species is only known from the type locality on South Gigante Island (Fig. 2). The snails were collected on shaded karst limestone.

##### Etymology.

The specific epithet lens (Latin *lentil*) refers to the shape of the new species. It is to be used as a noun in apposition.

#### Aulacospira mucronata

Taxon classificationAnimaliaStylommatophoraHypselostomatidae

(Möllendorff, 1887)

Figures 1E , 5J

Helix mucronata Möllendorff, 1887: 276, plate 8, figs 13–13b.
Aulacospira (Micropetasus) mucronata Möllendorff, 1890: 225.
Aulacospira mucronata Möllendorff, 1898: 150; Pilsbry 1917 (1916–1918): 222, plate 38, fig. 1.
Aulacospira (Aulacospira) mucronata Zilch, 1984: 167, plate 2, fig. 26.

##### Type locality.

“ad cacumina montis Licos et Uling insulae Cebu”.

##### Diagnosis.

Shell discoid and sharply keeled, body whorl (mainly the last half whorl) with slight subsutural furrow on the dorsal side, protoconch finely striated spirally, teleoconch roughly wrinkled and finely striated spirally; aperture without barriers, free from penultimate whorl.

##### Measurements (in mm).

H = 1.3–1.4, D = 3.1–3.3 (n = 3).

**Types examined**: Philippinen: Mte. Licos, Cebu, coll. Möllendorff, SMF 4609 (lectotype, selected by Zilch [1984], H: 1.4 mm, D: 3.2 mm); same data, coll. O Boettger excoll. Möllendorff, SMF 63880/3 paralectotypes; same data, coll. Möllendorff, SMF 4671(?)/7 paralectotypes.

##### Additional material examined.

Cebu Id., Tuburan Municipality, leg. HG Lee, ex GD Robinson; WF Webb, UF 110445 (4 shells); Cebu Id., leg. Univ. Alabama, M Smith Coll (MS-8513), UF 112319 (2 shells); Cebu Id., leg. Univ. Alabama, TH Aldrich Coll., Ex: WF Webb (THA-3080), UF 112318 (5 shells).

##### Distribution.

This species is only known from the type locality on Cebu Island (Fig. 2).

#### Aulacospira scalatella

Taxon classificationAnimaliaStylommatophoraHypselostomatidae

(Möllendorff, 1888)

Figures 3B , 5H

Helix scalatella Möllendorff, 1888: 145.
Aulacospira scalatella Möllendorff, 1898: 150; Pilsbry 1917 (1916–1918): 223, plate 38, figs 8–9; Schileyko 1998: 140, fig. 160.
Aulacospira (Aulacospira) scalatella Zilch, 1984: 167, plate 2, fig. 24.

##### Type locality.

“prope vicum Antipolo provinciae Manila”.

##### Diagnosis.

Shell flattened, and keeled, penultimate whorl elevated from body whorl resulting in a step-like appearance, body whorl with slight subsutural furrow on the dorsal side, protoconch spirally striated near teleoconch, teleoconch roughly wrinkled and finely striated spirally; aperture with four denticles (2 palatal, 1 parietal, 1 columellar), sometimes with an additional weak basal tooth; aperture free from penultimate whorl.

**Figure 3.**
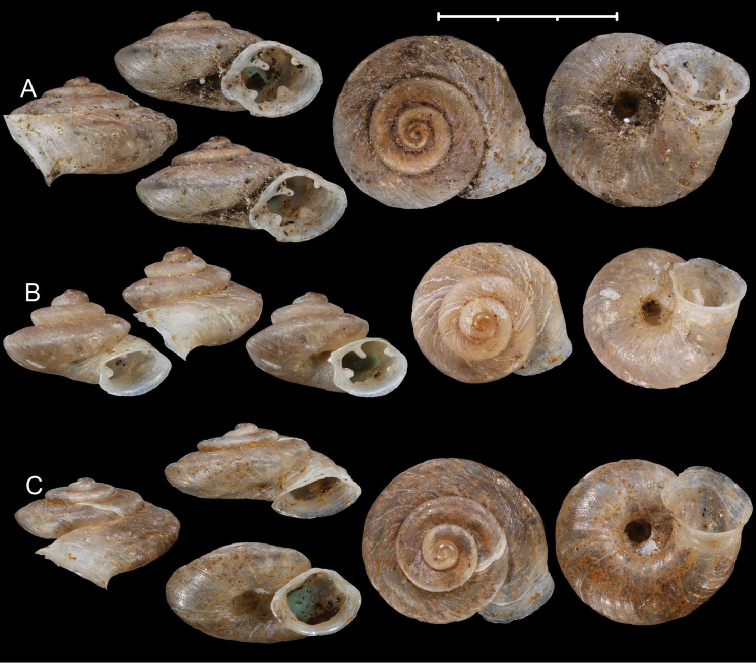
Shells of *Aulacospira* Möllendorff, 1890 species. **A***Aulacospirarhombostoma* Quadras & Möllendorff, 1896 (SMF 4612, lectotype) **B***Aulacospirascalatella* (Möllendorff, 1888) (SMF 4606, lectotype) **C***Aulacospiraporrecta* Quadras & Möllendorff, 1894 (SMF 4614, lectotype). Scale bar: 3 mm.

##### Measurements (in mm).

H = 1.6–1.8, D = 2.7–2.8 (n = 3).

##### Types examined.

Philippinen: Antipolo, Prov. Manila, Luzon, coll. Möllendorff, SMF 4606 (lectotype, selected by Zilch [1984], H: 1.8 mm, D: 2.8 mm); same data, SMF 4607/9 paralectotypes.

##### Additional material examined.

Luzon Id., Rizal Prov, mts of Bosoboso, leg. Univ Alabama, TH Aldrich Coll (THA-3081), UF 112322 (2 shells).

##### Distribution.

This species is known from two localities in Luzon island (Fig. 2).

#### Aulacospira porrecta

Taxon classificationAnimaliaStylommatophoraHypselostomatidae

Quadras & Möllendorff, 1894

Figures 3C , 5I

Aulacospira (Micropetasus) porrecta Quadras & Möllendorff, 1894: 95.
Aulacospira porrecta Möllendorff, 1898: 150; Pilsbry 1917 (1916–1918): 222–223, plate 38, fig. 2.
Aulacospira (Aulacospira) porrecta Zilch, 1984: 167, plate 2, fig. 27.

##### Type locality.

“in insula Ilin prope Mindoro”.

##### Diagnosis.

Shell strongly flattened and keeled, body whorl with slight subsutural furrow on the dorsal side, protoconch spirally striated, teleoconch roughly wrinkled and finely striated spirally; aperture with four denticles (2 palatal, 1 parietal, 1 columellar); aperture free from penultimate whorl.

##### Measurements (in mm).

H = 1.5–1.6, D = 2.8–3.3 (n = 3).

##### Types examined.

Philippinen: Insel Ilin b. Mindoro, coll. Möllendorff, SMF 4614 (lectotype, selected by Zilch [1984], H: 1.6 mm, D: 3.3 mm); same data, SMF 4615/5 paralectotypes.

##### Additional material examined.

Mindoro Id., leg. Univ Alabama, TH Aldrich Coll, ex: WF Webb (THA-3083), UF 112320 (1 shell).

##### Distribution.

This species is only known from the type locality (Ilin Island, Fig. 2).

#### Aulacospira rhombostoma

Taxon classificationAnimaliaStylommatophoraHypselostomatidae

Quadras & Möllendorff, 1896

Figures 3A , 5G

Aulacospira rhombostoma Quadras & Möllendorff, 1896: 8–9; Möllendorff 1898: 150; Pilsbry, 1917 (1916–1918): 223–224, plate 38, figs 13.
Aulacospira (Aulacospira) rhombostoma Zilch, 1984: 167, plate 2, fig. 28.

##### Type locality.

“in insula Tablas”.

##### Diagnosis.

Shell strongly flattened and keeled, body whorl with subsutural furrow on the dorsal side, protoconch smooth, teleoconch roughly wrinkled and finely striated spirally; aperture with a weak columellar and parietal tooth, and a palatal ridge corresponding to the furrow; aperture free from penultimate whorl.

##### Measurements (in mm).

H = 1.7–1.8, D = 3.2–3.5 (n = 3).

##### Types examined.

Philippinen, Tablas, coll. Möllendorff, SMF 4612 (lectotype, selected by Zilch [1984], H: 1.8 mm, D: 3.5 mm); same data, SMF 4613/7 paralectotypes.

##### Additional material examined.

Tablas Id., Looc, leg. Univ. Alabama, TH Aldrich Coll. (THA-3084), UF 112321 (4 shells).

##### Distribution.

This species is only known from the type locality on Tablas Island (Fig. 2).

#### Aulacospira triptycha

Taxon classificationAnimaliaStylommatophoraHypselostomatidae

Quadras & Möllendorff, 1895

Figures 1A , 5C

Aulacospira triptycha Quadras & Möllendorff, 1895: 76; Möllendorff 1898: 151.
Aulacospira triptycha Pilsbry, 1917 (1916–1918): 223.
Aulacospira (Aulacospira) triptycha Zilch, 1984: 167, plate 2, fig. 29.

##### Type locality.

“in monte Bathuan prope vicum Palanoc insulae Masbate”.

##### Diagnosis.

Shell discoid, strongly keeled, last half whorl with slight subsutural furrow on the dorsal side, protoconch smooth, teleoconch roughly wrinkled and finely striated spirally; aperture with a columellar and a palatal tooth; aperture adnate to penultimate whorl.

##### Measurements (in mm).

H = 1.8, D = 4.5 (n = 1, holotype with damaged apex).

##### Types examined.

Philippinen: Bathuan, Palanoc, Ins. Masbate, coll. Möllendorff, SMF 4611/1 syntype.

##### Distribution.

This species is only known from the type locality on Masbate Island. Palanoc is the former capital of Masbate at the mouth of the Lumbang River (12°21.5'N, 123°34.5'E, Fig. 2).

#### Aulacospira

Taxon classificationAnimaliaStylommatophoraHypselostomatidae

sp. (juvenile)

##### Material examined.

Palawan Province, Calamianes Islands, Coron Id., Kabudao Lake, NE shore, 40 m, 18 May 1987, Palawan Exp. leg., KA-520, UF 116981 (1 juvenile shell).

#### Genus Pseudostreptaxis

Taxon classificationAnimaliaStylommatophoraHypselostomatidae

Möllendorff, 1890

Pseudostreptaxis Möllendorff, 1890: 225; Schileyko 1998: 140.

##### Type species.

Helix (Aulacospira) Azpeitiae Hidalgo, 1890 by monotypy.

##### Diagnosis.

Shell globular, body whorl rounded or with smoothed peripheral angle, penultimate whorl rounded or narrowed or keeled (resulting in a streptaxoid shell shape); protoconch spirally striated, teleoconch with fine spiral striation and irregular growth wrinkles; aperture adnate to penultimate whorl, parietal callus weak, peristome almost discontinuous; apertural barriers 1–5; umbilicus narrow or relatively wide.

##### Remarks.

This genus has been monotypic so far. The type species, *Pseudostreptaxisazpeitiae* is characterized by a shell shape having distorted upper whorls reminiscent of those of Streptaxidae, and 4–5 apertural barriers. *Pseudostreptaxisharli* sp. n. differs conspicuously from *P.azpeitiae* by having a single parietal tooth and possessing a globular shell, but it often has a narrowed penultimate whorl similar to that of *P.azpeitiae*. Since the two species of *Pseudostreptaxis* differ considerably from each other, a modified diagnosis must be given. Due to the rounded body whorl this genus is treated as a full genus, as did to Schileyko (1998). Although this morphological difference might not seem to be sufficient for genus-level distinction, we rather use the most recent taxonomy.

#### Pseudostreptaxis azpeitiae

Taxon classificationAnimaliaStylommatophoraHypselostomatidae

(Hidalgo, 1890)

Figures 4A , 5A

Helix (Aulacospira) Azpeitiae Hidalgo, 1890: 120.
Aulacospira azpeitiae Möllendorff, 1898: 151.
Aulacospira (Pseudostreptaxis) azpeitiae Pilsbry, 1917 (1916–1918): 224, plate 38, figs 14, 17; Zilch 1984: 167–168.
Pseudostreptaxis azpeitiae Schileyko, 1998: 141, fig. 161.

##### Type locality.

“Isla Catanduanes”.

##### Diagnosis.

Shell streptaxoid (penultimate whorl keeled, rapidly descending toward aperture and oblique to shell axis; body whorl rather rounded, descending more slowly); protoconch with very weak (barely visible) spiral striation, teleoconch with rough wrinkles and some spiral striation; aperture with five teeth (1 parietal, 2 palatal, 1 basal that can be absent sometimes, 1 columellar).

**Figure 4.**
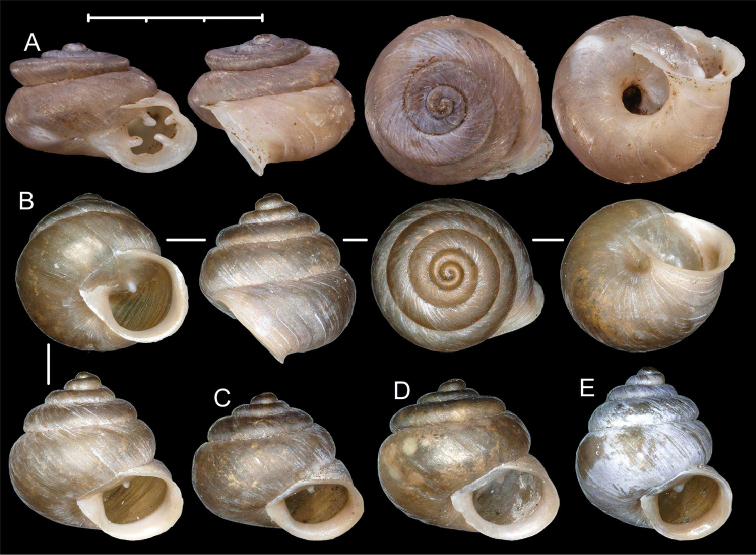
Shells of *Pseudostreptaxis* Möllendorff, 1890 species. **A***Pseudostreptaxisazpeitiae* (Hidalgo, 1890), SMF 63882 **B***Pseudostreptaxisharli* sp. n., holotype (NHMW 113033) **C–E***Pseudostreptaxisharli* sp. n., paratypes from the same sample (NHMW 113034). Scale bar: 3 mm.

**Figure 5.**
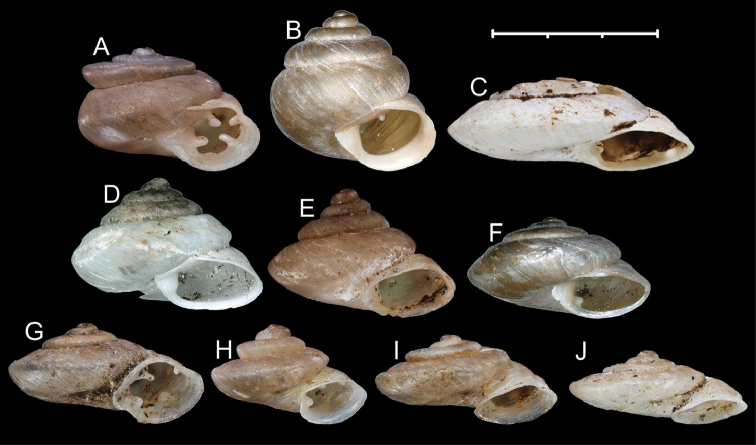
Synoptic view of *Aulacospira* Möllendorff, 1890 and *Pseudostreptaxis* Möllendorff, 1890 species. **A***Pseudostreptaxisazpeitiae***B***Pseudostreptaxisharli* sp. n. **C***Aulacospiratriptycha***D***Aulacospirakrobyloides* sp. n. **E***Aulacospirahololoma***F***Aulacospiralens* sp. n. **G***Aulacospirarhombostoma***H***Aulacospirascalatella***I***Aulacospiraporrecta***J***Aulacospiramucronata*. Scale bar: 3 mm.

##### Types.

Not examined.

##### Additional material examined.

Philippinen: Busuanga (Calamianes), Bintuan, coll. Möllendorff, SMF 63882/9; Palawan Province, Calamianes Ids., Busuanga Id., leg. Beal-Maltbie Coll., UF 240009 (3 shells); Palawan Province, Calamianes Ids., Busuanga Id., Penon de Bintuan, leg. Univ. Alabama, T.H. Aldrich Coll, ex: WF Webb (THA-3082), UF 112316 (2 shells).

##### Distribution.

The type locality of this species is Catanduanes Island, which is situated on the eastern edge of the Philippines. However, all other material of this species was collected on Busuanga Island (Calamianes) in the western part of the Philippine Islands. Möllendorff (1898) gives only Busuanga as the distribution without comment. A hypselostomatid species occurring on two islands nearly 500 km apart is highly unlikely, given that all other members of this family are narrow-range endemics. Furthermore, the newly described congeneric, *P.harli* sp. n. (see below), also occurs geographically near the Calamianes. Therefore, we conclude that the occurrence of *P.azpeitiae* on Catanduanes is probably erroneous, and this species only occurs on Busuanga Island.

#### Pseudostreptaxis harli

Taxon classificationAnimaliaStylommatophoraHypselostomatidae

Páll-Gergely & Schilthuizen sp. n.

http://zoobank.org/7EE8FD8F-207C-4232-B191-92DE71936462

Figures 4B–E , 5B

##### Type material.

Palawan Province, El Nido Municipality, Shimizu Island, Panaustard (?) leg. J Harl, 15 Feb 2009, NHMW 113033 (holotype); same data, NHMW 113034/3 photographed paratypes (Fig. 4C–E), HNHM 103477/5 paratypes; PGB/10 paratypes, SMF 351762/3 paratypes; REI/43 paratypes; Palawan Province, El Nido Municipality, Miniloc Island, 11.146N, 119.313E, sieved from litter underneath limestone cliff, leg. M Schilthuizen, 18 Mar 2018, TxEx-PA0003-45, RMNH.MOL.340315/30 paratypes, PGB/60 paratypes.

##### Diagnosis.

Shell globular, protoconch spirally striated, teleoconch roughly wrinkled and spirally striated; aperture with a single parietal tooth that sometimes consists of two tubercles; aperture adnate to penultimate whorl.

##### Description.

Shell globular (0.95–1.15 times as wide as high) with bulging whorls and deep suture; in some specimens penultimate whorl narrowed, somewhat similarly to that of *Pseudostreptaxisazpeitiae*; colour light brownish-greyish to dark purple; entire shell consists of 4–4.5 whorls; protoconch consisting of ca. 1.25 whorl, finely granulose and spirally striated; teleoconch finely granulose, with some fine, irregular wrinkles and with dense, hardly visible spiral striation; aperture oblique to shell axis, aperture rounded; peristome thickened and expanded, especially in the basal and columellar direction; parietal callus weak, appears as a lime layer on the penultimate whorl; parietal tooth (lamella) elevated, deeply situated, relatively long, in some specimens consisting of two tubercles, which might indicate that it is homologous with the parieto-angular lamella; no other apertural barriers present; umbilicus open but very narrow, showing only the last whorl, mostly covered by expanded peristome.

##### Measurements (in mm).

H = 2.6–3.0, D = 2.9–3.1 (n = 2).

##### Differential diagnosis.

*Pseudostreptaxisazpeitiae* has a keeled penultimate whorl resulting in a shell shape reminiscent of the Streptaxidae. Moreover, the new species has a single parietal tooth, while *Pseudostreptaxisazpeitiae* possesses 4–5 apertural barriers.

##### Remarks on shell shape.

This new species is placed in *Pseudostreptaxis* because of the rounded body whorl, which is keeled in *Aulacospira*.

**Figure 6.**
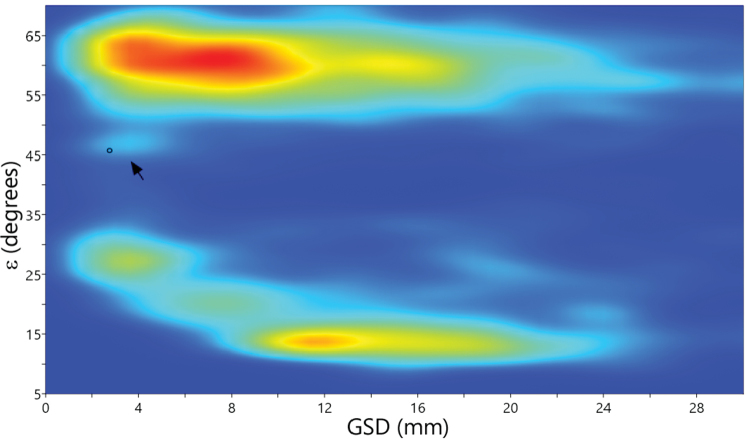
Kernel density distribution of shell shape angles (ε) against the mean greater shell dimensions (GSD) for 2134 pulmonate genera. The kernel density increases from blue through green and yellow to red colours. Arrow points at the diffuse cluster of approximately 30 roughly equiaxial genera within the approximate ranges of ε = 44°–49° and GSD = 1.5–5 mm and within which *P.harli* sp. n. (circle) is located.

The shell of *P.harli* sp. n. is unusually “spherical”. To obtain a uniform linear distribution, the shell shape may be expressed as the angle obtained by taking the inverse tangent of the ratio D/H (Örstan 2018). The distribution of the shell shape angles (ε) thus obtained against the mean greater shell dimensions (GSD, the larger of the shell height or diameter) for 2134 pulmonate genera (Fig. 6) forms one major cluster of flat (ε > 50°) shells and at least three smaller clusters of tall (ε < 40°) shells. In addition, there is a diffuse cluster of approximately 30 roughly equiaxial and small genera within the approximate ranges of ε = 44°–49° and GSD = 1.5–5 mm (Fig. 6, arrow). The mean shell values (ε = 46°, GSD = 3.03 mm) for *Pseudostreptaxisharli* sp. n. place this species in the latter cluster. The genera in this cluster, being members of eight families, form a phylogenetically diverse group. The evolutionary convergence of these small genera on a similar shell shape suggests that they and any individual species that may be included in this cluster, including *P.harli* sp. n., share a unique biological trait that has enabled them to overcome the opposing selective forces that have prevented more species to evolve equiaxial shell shapes.

##### Distribution.

This species is known from two adjacent tiny islands (Miniloc and Shimizu) on the northeastern coast of Palawan (Fig. 2).

##### Etymology.

This new species is named after Josef Harl, a friend of the first author, who first collected this new species and provided it for study.

## Supplementary Material

XML Treatment for Aulacospira

XML Treatment for Aulacospira hololoma

XML Treatment for Aulacospira krobyloides

XML Treatment for Aulacospira lens

XML Treatment for Aulacospira mucronata

XML Treatment for Aulacospira scalatella

XML Treatment for Aulacospira porrecta

XML Treatment for Aulacospira rhombostoma

XML Treatment for Aulacospira triptycha

XML Treatment for Aulacospira

XML Treatment for Pseudostreptaxis

XML Treatment for Pseudostreptaxis azpeitiae

XML Treatment for Pseudostreptaxis harli
